# Effect of premature rupture of membranes on preterm labor: a case-control study in Cilegon, Indonesia

**DOI:** 10.4178/epih.e2020025

**Published:** 2020-04-10

**Authors:** Ita Marlita Sari, Asri C. Adisasmita, Sabarinah Prasetyo, Dwirani Amelia, Ratih Purnamasari

**Affiliations:** 1 Faculty of Medicine, Sultan Ageng Tirtayasa University, Banten, Indonesia; 2 Epidemiology Department, Public Health Faculty, University of Indonesia, Depok, Indonesia; 3 Biostatistic Department at Public Health Faculty, University of Indonesia, Depok, Indonesia; 4 Obstetrician at Budi Kemuliaan Hospital, Jakarta, Indonesia; 5 Cilegon Public Hospital, Banten, Indonesia

**Keywords:** Obstetric labor, Premature birth, Rupture, Pregnant women, Case control studies, Indonesia

## Abstract

**OBJECTIVES:**

The global prevalence of preterm labor is approximately 11.1% of live births. However, preterm labor contributes to 75-80% of neonatal morbidity and mortality. The morbidity experienced by preterm infants may continue to influence their subsequent development, imposing physical, psychological, and economic burdens. Premature rupture of membranes (PROM) is a causal factor that may affect preterm birth. Previous studies have shown an association between PROM and preterm labor, but this association should be investigated in more diverse populations. Therefore, this study was conducted in Cilegon, Indonesia to determine the magnitude of the risk of preterm labor associated with PROM at Cilegon Hospital from July 2014 to December 2015.

**METHODS:**

This case-control study used data from patients’ medical records. The cases were all mothers who delivered at less than 37 weeks of gestation, while the control population comprised all mothers who delivered at greater or equal to 37 weeks. The data were analyzed using logistic regression.

**RESULTS:**

The bivariate analysis yielded an odds ratio (OR) of 2.97 (95% confidence interval [CI], 1.92 to 4.59) before controlling for covariates. The model derived through multiple regression analysis after controlling for education, history of preterm labor, and anemia resulted in an OR of 2.58 (95% CI, 1.68 to 3.98).

**CONCLUSIONS:**

Mothers who experience PROM during pregnancy were at a 2.58 times higher risk of preterm labor after controlling for education, history of preterm labor, and anemia.

## INTRODUCTION

The reduction in neonatal mortality targeted as part of the Millennium Development Goals has not yet been fully achieved. An important barrier to progress toward achieving this goal is death associated with preterm birth [1]. Preterm childbirth refers to the delivery or birth of a baby with a gestational age of less than 37 weeks, in which gestational age is calculated based on the mother’s last menstrual period [2]. Preterm labor accounts for as much as 75-80% of neonatal morbidity and mortality [3]. Preterm infant morbidity imposes physical, psychological, and economic burdens on babies, mothers, and families. Globally, approximately 11.1% of live births are preterm deliveries [1,2,4-6].

The rate of preterm labor in developing (low-income/middle-income) countries is higher than that in developed (high-income) countries [7]. In low-income/middle-income countries, more than 60% of preterm deliveries occur in Africa and South Asia [1,7]. The preterm birth rate at a hospital in southern India in 2014 was 5.8% [8], while the rate of preterm birth at Cipto Mangunkusumo Hospital Jakarta in 2013 was 38.5% [9].

An important factor associated with preterm birth is premature rupture of membranes (PROM). Approximately 25-40% of cases of preterm labor occur due to PROM [10,11]. The frequency of PROM among patients experiencing preterm labor at a western Iranian hospital in 2014 was 52.8% [12]. A key variable related to PROM is the latency period (LP), which refers to the time between the onset of PROM and labor [13]. A long LP can increase the risk of perinatal death and amnionitis [14,15]. When PROM occurs with an LP of more than 24 hours, the risk of perinatal death increases, and the risk of amnionitis also increases to more than 50%. The pathogenesis of PROM is unclear, but it is thought to relate to intrapartum infections and associated biochemical changes that occur in the collagen of the extracellular matrices of the amnion and the chorion, as well as fetal membrane apoptosis [5,16].

A study conducted in a western Iranian hospital found that pregnant women with PROM have a risk of preterm labor of 2.65 (95% confidence interval [CI], 1.44 to 4.85) relative to women without PROM [12]. The present study was conducted at Cilegon Hospital because the prevalence of preterm birth had increased in that facility in 2014. The objective of this study was to assess the risk of preterm labor associated with PROM at Cilegon Hospital for the period between July 2014 and December 2015.

## MATERIALS AND METHODS

**Figure 1.**
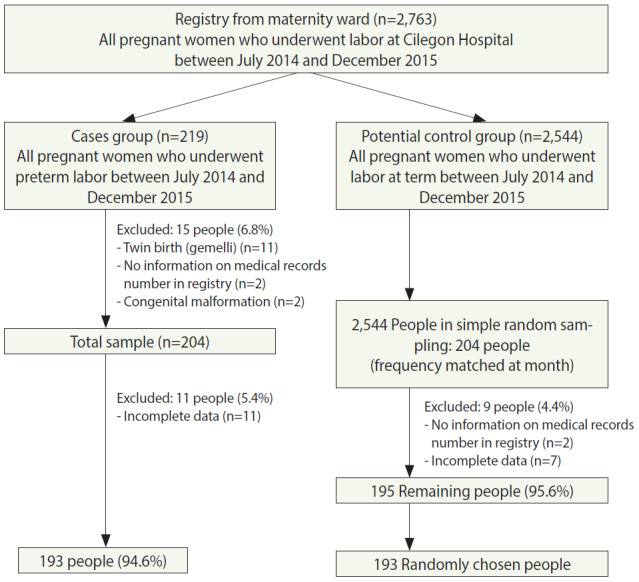
Sampling Process.

This study used a case-control design because the incidence of preterm labor was relatively low. Apart from PROM, other variables studied included maternal characteristics (age, education, and work status), previous obstetric history (history of abortion and history of preterm labor), and variables related to the current pregnancy (parity, duration from previous delivery, usage of antenatal care, maternal anemia, hypertension of pregnancy, maternal history of other diseases, and antepartum hemorrhage). The rate of preterm delivery at Cilegon Hospital was 5.1% in 2013 and increased to 5.4% in 2014. The study subjects were pregnant women who experienced preterm labor at Cilegon Hospital between July 2014 and December 2015. The control subjects were pregnant women who experienced at-term labor at Cilegon Hospital during the same interval. As shown in Figure 1, the case group consisted of 193 respondents after exclusion, so the control group was similarly designed to contain 193 subjects, and the total number of subjects was 386. Cases were identified via total sampling, while the controls were selected by simple random sampling and frequency matching by month. The researchers created data collection forms that were filled out by enumerators to align with the data from medical records. However, the enumerators were not blinded to the outcome variable during data retrieval.

The data were subjected to univariate analysis to describe the characteristics and comparability of the cases and controls. Then, bivariate analysis was used to assess the relationship between the dependent and independent variable via the chi-square test. In addition, odds ratios (ORs) and CIs were calculated for the risk factors.

A multiple analysis logistic regression model was then used to assess the relationship between the independent variable and the dependent variable while controlling for several covariates. Multiple analysis consisted of the modeling phase (crude modeling), full modeling, a confounding assessment, and final (fixed) modeling. After development of the crude model, bivariate analysis was carried out for each potential confounding variable. When the results of bivariate analysis yielded a p-value < 0.25, that variable was entered into the full model. If p-value > 0.25, but the variable was determined to be sufficiently important, the variable was included in the full model. Variables that yielded p-values < 0.05 in the full model were entered into the fixed model. This final model was the most fitting and parsimonious model of the results after controlling for potential confounding relationships by using the Stata version 12 (StataCorp., College Station, TX, USA).

### Ethics statement

The research data came from secondary data of medical records of patients in Cilegon Public Hospital. All data used in this study will be kept the confidentiality of the subject’s identity and the confidentiality of the data is only for research purposes. The research protocol was submitted to the Research Ethics Commission Faculty of Public Health University of Indonesia.

## RESULTS

The sampling process is shown in Figure 1. Data collection from the maternity ward register book yielded information on 2,763 deliveries that took place between July 2014 and December 2015, including 219 potential case deliveries and 2,544 potential control deliveries. Some pregnant women were excluded from the case group, resulting in a final sample of 193 cases. In the control group, after exclusion, simple random sampling and frequency matching by month yielded a control group of 193 mothers. The overall prevalence of preterm labor at Cilegon Hospital was 7.9% for the period from July 2014 to December 2015. The overall rate of PROM in women with preterm labor was 66.3%, whereas the rate of PROM in women with at-term labor was 39.9%. Of the mothers who did exhibit PROM, both mothers with preterm labor (56.2%) and those with at-term labor (66.3%) tended to have an LP of < 12 hours. The power of this study was about 100%. Table 1 shows the distribution of maternal characteristics, obstetric history, and variables related to the current pregnancy among the case and control subjects. Overall, most of the mothers were 20-35 years old and had a high school education or equivalent. Among the case subjects, the majority of mothers were not working (50.6%), while the majority of subjects in the control group were working mothers (54.5%). Most mothers had no history of abortion or previous preterm labor, both among case and control subjects. With regard to the current pregnancy, in both groups, most mothers were multiparous, had an interval of more than 24 months since the previous delivery, never received antenatal care, did not have hypertension in pregnancy, had no history of other diseases, and did not have antepartum bleeding. Mothers with preterm labor were relatively likely to have anemia (52.3%), while women with labor at term were relatively likely to not have anemia (71.0%).

The relationships of PROM, maternal characteristics, obstetric history, and current pregnancy-related variables with preterm labor are shown in Table 2. Table 2 includes values for crude ORs, full-model ORs, and fixed-model ORs obtained via multiple regression analysis. The final (fixed) model accounted for the confounding variables of maternal education, history of preterm labor, and maternal anemia. Mothers who had elementary school-equivalent education displayed a risk of preterm labor of 1.70 (95% CI, 1.00 to 2.89) relative to women who were more educated (that is, those who had a junior high, high school, or university education). The history of preterm labor was divided into primiparity (1 previous preterm labor) and multiparity (> 1 previous preterm labor). Primiparous preterm mothers had a risk of preterm labor of 9.89 (95% CI, 2.14 to 45.64) relative to multiparous preterm mothers. This variable had a relatively wide 95% CI and a relatively large OR. Based on the data shown in Table 1, it can be inferred that these findings may have occurred because the case group did not have a sufficient sample size. Mothers with anemia had a risk of preterm labor of 2.49 (95% CI, 1.60 to 3.88) compared to mothers who were not anemic.

The relationship between the PROM LP and the risk of preterm labor is shown in Table 3. The final (fixed) model showed that women with a PROM LP of < 12 hours had a risk of preterm labor of 2.11 (95% CI, 1.29 to 3.45) relative to women who did not have PROM. In contrast, women with a PROM LP of > 12 hours had a preterm labor risk of 3.55 (95% CI, 2.00 to 6.29) compared with women who did not have PROM. These results indicated a dose-dependent relationship, in that a longer LP increased the risk of preterm labor.

The fitted model obtained via multiple analysis after controlling for education, history of preterm labor, and anemia status found that mothers who had PROM during pregnancy had a risk of preterm labor that was 2.58 times higher (95% CI, 1.68 to 3.98) than that of mothers without PROM.

## DISCUSSION

The objective of this study was to describe the relationship between PROM and preterm labor, and specifically to determine the risk of preterm labor in pregnant women with PROM. As shown via multiple regression analysis, the association between PROM and preterm labor was significant, with an OR of 2.58 (95% CI, 1.63 to 3.98). The CI for this finding was relatively narrow, implying that chance variations had little impact. Additionally, the power of this study was about 100.

The limitation of this study was using secondary data from patients’ medical records, so the study variables had to be adapted to the data available in the medical records. In general, almost all variables could be obtained sufficiently through the medical records. Unfortunately, however, these records did not include data regarding body mass index (kg/m^2^), smoking history, economic status, or physical status.

Given that the dependent variable was contingent upon routine data recording, any errors may have resulted in selection bias in either the case or the control group. Selection bias was minimized by selecting control cases from the same source population. Additionally, the case sample was selected via total sampling and the control sample via simple random sampling. The missing data in this study comprised less 10% of the total data, further reducing selection bias.

Differential misclassification bias may have been introduced by the researcher’s knowledge of the outcome variable. The researcher attempted to minimize bias by collecting data from a registry with the help of an enumerator. However, during data collection, the researcher was not blinded to the outcome or to the main independent variable. In contrast, non-differential misclassification bias may have occurred due to measurement-related difficulties, which may have been caused by inaccurate definitions of the exposure and the outcome.

Frequency matching was performed to ensure comparability between the case and control groups. Confounding variables were controlled for via multiple analysis. Besides, reaserchers found a dose-response relationship between the LP of PROM and the occurrence of preterm labor. In particular, the longer the LP of PROM, the higher the risk of preterm labor. Preterm labor can be affected by many factors, and the theoretical biological mechanism of preterm labor has been clearly outlined.

The participation rate of the sample was quite high, so the results can be generalized to the eligible source population, which comprised 2,763 people in total. Only a few cases were excluded, further emphasizing that the results can be applied to the source population. However, this was a hospital-based study, so it may be difficult to generalize to other populations.

A biological mechanism that explains the relationship of PROM with the onset of preterm labor involves intrapartum infection that activates pro-inflammatory cytokines (interleukin [IL]-1, tumor necrosis factor alpha, and IL-6), then stimulates the maternal decidua and the fetal membrane to release metalloproteinase matrix enzymes/proteases and prostaglandins. Then, in concert, the thinning of the cervix and the promotion of uterine contractions stimulate preterm labor.

## Figures and Tables

**Table 1.** Distribution of maternal characteristics, obstetric history, variables related to current pregnancy, and premature rupture of membranes (PROM) among cases and controls

Variables	Case	Control	p-value
Maternal age (yr)			
<20	14 (7.2)	9 (4.7)	0.407
20-35	154 (79.8)	162 (83.9)	
>35	25 (12.9)	22 (11.4)	
Education			
University/academy	16 (8.3)	24 (12.4)	0.156
High school/equivalent	80 (41.4)	79 (40.9)	
Junior high school/equivalent	51 (26.4)	55 (28.5)	
Elementary school/equivalent	46 (23.8)	35 (18.1)	
Work status			
Working	20 (45.4)	24 (54.5)	
Not working	173 (50.6)	169 (49.4)	0.522
Abortion history			
No	164 (85.0)	160 (82.9)	
Yes	29 (15.0)	33 (17.1)	0.579
Preterm labor history			
Primiparity (had 1)	12 (70.6)	0 (0.0)	
Multiparity (had >1)	5 (29.4)	2 (100)	<0.001
Parity			
Multiparous	108 (56.0)	119 (61.7)	
Primiparous	85 (44.0)	74 (38.3)	0.255
Interval since previous labor (mo)			
<18	9 (4.7)	3 (1.5)	
18-24	9 (4.7)	6 (3.1)	0.051
>24	175 (90.7)	184 (95.3)	
Antenatal care			
Yes	148 (76.7)	155 (80.3)	
No	45 (23.3)	38 (19.7)	0.386
Hypertension in pregnancy			
No	158 (81.9)	150 (77.7)	
Yes	35 (18.1)	43 (22.3)	0.311
History of other diseases^1^			
No	171 (88.6)	173 (89.6)	
Yes	22 (11.4)	20 (10.4)	0.744
Maternal anemia			
No anemia	92 (47.7)	137 (71.0)	
Anemia	101 (52.3)	56 (29.0)	<0.001
Antepartum bleeding			
No	184 (95.3)	189 (97.9)	
Yes	9 (4.7)	4 (2.1)	0.158
PROM			
No	65 (33.7)	116 (39.9)	
Yes	128 (66.3)	77 (60.1)	<0.001

Values are presented as number (%).

1 Hypertension, tuberculosis, diabetes mellitus, hepatitis, tumor/cancer, heart disease, asthma, other infection.

**Table 2.** Association of premature rupture of membranes (PROM), maternal characteristics, obstetric history, and current condition of pregnancy with preterm labor^1^

Variables	Crude	p-value	Full-model	p-value^2^	Fixed-model	p-value^3^
PROM without latency period						
No PROM	1.00 (reference)		1.00 (reference)		1.00 (reference)	<0.001
PROM	2.97 (1.92, 4.59)	<0.001	2.72 (1.74, 4.25)	<0.001	2.58 (1.68, 3.98)	
Maternal age (yr)						
<20	1.64 (0.69, 3.90)	0.262	-		-	
20-35	1.00 (reference)		-		-	
>35	1.20 (0.57, 2.21)	0.569	-		-	
Education						
University/high school/junior high school	1.00 (reference)		1.00 (reference)		1.00 (reference)	
Elementary school or equivalent	1.41 (0.84, 2.39)	0.169	1.99 (1.14, 3.44)	0.015	1.70 (1.00, 2.89)	0.045
Work status						
Not working	1.00 (reference)		-		-	
Working	0.81 (0.43, 1.53)	0.522	-		-	
History of abortion						
No	1.00 (reference)		-		-	
Yes	0.86 (0.48, 1.53)	0.579	-		-	
History of preterm labor						
Primiparity (had 1)	10.93 (2.47, 99.08)	<0.001	9.20 (1.95, 43.32)	0.005	9.89 (2.14, 45.64)	0.003
Multiparity (had >1)	1.00 (reference)		1.00 (reference)		1.00 (reference)	
Parity						
Multiparous	1.00 (reference)		1.00 (reference)		-	
Primiparous	1.27 (0.83, 1.94)	0.244	1.39 (0.87, 2.21)	0.169	-	
Interval since previous labor (mo)						
<18	3.15 (0.83, 11.93)	0.074	2.31 (0.49, 10.81)	0.289	-	
18-24	1.58 (0.55, 4.53)	0.394	0.53 (0.15, 1.94)	0.341	-	
>24	1.00 (reference)		1.00 (reference)		-	
Antenatal care						
Yes	1.00 (reference)		-		-	
No	1.24 (0.74, 2.08)	0.386	-		-	
Hypertension in pregnancy						
No	1.00 (reference)		-		-	
Yes	0.77 (0.45, 1.31)	0.311	-		-	
History of other diseases^4^						
No	1.00 (reference)		-		-	
Yes	1.11 (0.56, 2.23)	0.744	-		-	
Maternal anemia						
No anemia	1.00 (reference)		1.00 (reference)		-	
Anemia	2.69 (1.73, 4.18)	<0.001	2.33 (1.49, 3.66)	<0.001	-	
Antepartum bleeding						
No	1.00 (reference)		1.00 (reference)		1.00 (reference)	
Yes	2.31 (0.63, 10.43)	0.158	3.10 (0.86, 11.13)	0.083	2.49 (1.60, 3.88)	<0.001

Values are presented as odds ratio (95% confidence interval).

1 Multiple regression analysis consisted of a modeling phase (crude modeling), a full model phase, a confounding assessment, and the final (fixed) model.

2 Significant at p<0.25 (or p>0.25 for a variable considered sufficiently important) and analyzed in the full model.

3 Significant at p<0.05 and analyzed in the fixed model.

4 Hypertension, tuberculosis, diabetes mellitus, hepatitis, tumor/cancer, heart disease, asthma, other infection.

**Table 3.** Association between LP of PROM and preterm labor^1^

Variables	Crude	p-value	Full-model	p-value^2^	Fixed-model	p-value^3^
PROM without LP						
No PROM	1.00 (reference)		1.00 (reference)		1.00 (reference)	
PROM	2.97 (1.92, 4.59)	<0.001	2.72 (1.74, 4.25)	<0.001	2.58 (1.68, 3.98)	<0.001
PROM with LP (hr)						
No PROM	1.00 (reference)		1.00 (reference)		1.00 (reference)	
PROM LP <12	2.52 (1.55, 4.08)	0.003	2.21 (1.33, 3.65)	0.003	2.11 (1.29, 3.45)	0.003
PROM LP >12	3.84 (2.14, 6.89)	<0.001	3.80 (2.10, 6.89)	<0.001	3.55 (2.00, 6.29)	<0.001

Values are presented as odds ratio (95% confidence interval). PROM, premature rupture of membranes; LP, latency period.

1 Values indicate the presence of a dose-response relationship in that a longer LP is associated with an increased risk of preterm labor; Multiple analysis consisted of a modeling phase (crude modeling), a full model phase, a confounding assessment, and the final (fixed) model.

2 Significant at p<0.25 (or p>0.25 for a variable considered sufficiently important) and analyzed in the full model.

3 Significant at p<0.05 and analyzed in the fixed model.
